# Bone Tissue Changes in Individuals Living with HIV/AIDS: The Importance of a Hierarchical Approach in Investigating Bone Fragility

**DOI:** 10.3390/jpm14080791

**Published:** 2024-07-26

**Authors:** Jelena Jadzic, Gordana Dragovic, Relja Lukic, Bozana Obradovic, Marija Djuric

**Affiliations:** 1 Center of Bone Biology, Faculty of Medicine, University of Belgrade, 11000 Belgrade, Serbia; marija.djuric@med.bg.ac.rs; 2 Department of Pharmacology, Clinical Pharmacology and Toxicology, Faculty of Medicine, University of Belgrade, 11000 Belgrade, Serbia; gordana.dragovic@med.bg.ac.rs (G.D.); bozana.dimitrijevic@med.bg.ac.rs (B.O.); 3 Faculty of Medicine, University of Belgrade, 11000 Belgrade, Serbia; relja.lukic@med.bg.ac.rs; 4 Obstetrics and Gynecology Clinic “Narodni Front”, 11000 Belgrade, Serbia

**Keywords:** HIV, PLWHIV, bone fracture, bone strength, bone mineral density, hierarchical bone organization, antiretroviral therapy

## Abstract

Skeletal alterations and their complications can significantly impact the quality of life and overall prognosis of patients living with HIV (PLWHIV). Considering skeletal alterations are often asymptomatic and unapparent during routine clinical evaluation, these conditions are frequently overlooked in the clinical management of PLWHIV. However, since the use of combined antiretroviral therapy (cART) has increased life expectancy in PLWHIV effectively, osteopenia, osteoporosis, and bone fragility are now considered to have a major health impact, with a substantial increase in healthcare costs. This narrative literature review aimed to provide a comprehensive overview of the contemporary literature related to bone changes in PLWHIV, focusing on the importance of taking a multi-scale approach in the assessment of bone hierarchical organization. Even though a low bone mineral density is frequently reported in PLWHIV, numerous ambiguities still remain to be solved. Recent data suggest that assessment of other bone properties (on various levels of the bone structure) could contribute to our understanding of bone fragility determinants in these individuals. Special attention is needed for women living with HIV/AIDS since a postmenopausal status was described as an important factor that contributes to skeletal alterations in this population. Further research on complex etiopathogenetic mechanisms underlying bone alterations in PLWHIV may lead to the development of new therapeutic approaches specifically designed to reduce the health burden associated with skeletal disorders in this population. A major challenge in the clinical management of PLWHIV lies in the adverse skeletal effects of some frequently prescribed cART regimens (e.g., regimens containing tenofovir disoproxil fumarate), which may require a switch to other pharmacological approaches for maintained HIV infection (e.g., regimens containing tenofovir alafenamide). Taken together, the findings are indicative that the HIV/AIDS status should be taken into consideration when designing new guidelines and strategies for individualized prevention, diagnosis, and treatment of increased bone fragility.

## 1. Introduction

Since the development of combined antiretroviral therapy (cART), there has been a tendency toward an increasing percentage of people living with HIV/AIDS (PLWHIV) over the age of 50, which is accompanied by an increased risk of bone fracture in this population [1,2,3]. Recent meta-analyses reported at least a twofold increase in fragility fracture risk in PLWHIV compared to the general population [2,4]. However, the risk of fragility fracture displays a certain level of site specificity in PLWHIV, with the most prominent susceptibility to vertebral, femoral, and wrist fractures noted in these individuals [2,3,4]. Particularly worrying is the report on a substantial increase in fracture risk among PLWHIV aged between 25 and 54 [5], revealing a fracture risk shift to the younger (working age) population [6]. Also, it is important to note that special considerations are needed for women living with HIV/AIDS (WLWHIV) [7] since the postmenopausal status was described as an important factor that contributes to an increased fracture risk in this population [2,8]. Moreover, the association between certain cART regimens and the fragility fracture risk is intensively debated in the contemporary literature [6,9], warranting further research.

Given that the occurrence of bone fractures and their complications are preventable [10,11], it is important to understand all factors that contribute to increased bone fragility in PLWHIV. Addressing the issue of an increased fracture risk will improve quality of life, promote healthy aging, and reduce healthcare costs for these individuals. Considering the clinical relevance of bone fragility, this article aimed to provide a comprehensive narrative overview of the contemporary literature related to skeletal alterations in PLWHIV, with a particular focus on the importance of a multi-scale approach in assessing the bone hierarchical organization.

## 2. Literature Search Strategy

An electronic search was conducted using the PubMed/Medline, Cochrane, Web of Science, and National Library of Medicine—National Institutes of Health databases on 3 June 2024. To identify published articles on skeletal alterations in PLWHIV, two authors independently obtained search results using the following search terms: “HIV” OR “AIDS” OR “PLWHIV” OR “WLWHIV” AND “osteopenia” OR “osteoporosis” OR “bone fracture” OR “bone mineral density” OR “bone micro-architecture” OR “bone quality”. Two authors independently reviewed the obtained search results. Only preclinical human studies and clinical studies written in English were considered eligible to be included in this review. Studies on animal models and studies written in other languages were excluded from this review. Discrepancies were resolved through joint discussion, and all authors agreed with the final pool of articles included in this review.

## 3. Osteodensitometry Findings in PLWHIV

Since it is not always possible to directly assess bone fracture occurrence and bone fracture risk (especially in an individualized manner), modern studies rely on the measurement of various clinical surrogate endpoints of increased bone fragility [12]. The “gold standard” in the clinical assessment of bone fragility is the bone mineral density (BMD) measurement using dual-energy X-ray absorptiometry (DXA) [10]. It is defined as the bone mineral content (BMC) per analyzed bone area (B.Ar). Still, BMD alterations are most commonly expressed as a T score (which refers to the number of standard deviations above or below the mean BMD of a population of healthy female adults at the age of their peak bone mass). Based on the most recent World Health Organization definition, osteoporosis is a systemic skeletal disease characterized by low bone mass and micro-architectural deterioration, causing increased susceptibility to fragility fracture, defined as a T score ≤ −2.5. Osteopenia is diagnosed if T score values are between −1 and −2.5 [10,13]. Alternatively, the Z score refers to the number of standard deviations above or below the mean BMD of a population that is of the same sex and age as the investigated patient [14], meaning that Z scores are preferably used in children, young male patients, premenopausal women, and for diagnosis of secondary osteoporosis. The prevalence of osteoporosis among PLWHIV is reported to be very variable (ranging between 0% and 34%) [13,15]. Still, numerous studies consistently report that a low lumbar spine and hip BMD are present both in men and women living with HIV/AIDS [8,15,16,17,18,19], which was confirmed in recent meta-analyses [4,20,21]. The discrepancy in previous reports about the skeletal alterations in PLWHIV could at least partially be explained by heterogeneity in the study design, number of participants, age, weight, and gender of the participants, and presence of accompanying comorbidities. These cofounding effects point towards the reasoning that the underlying factors causing low BMD in PLWHIV are multifactorial, warranting further clarification in future well-designed, large, prospective clinical studies. The most important obstacle in the clinical management of bone changes in PLWHIV is their asymptomatic nature [22], leaving them unapparent and frequently overlooked during routine clinical evaluations. Thus, it is of most importance to include data about the HIV/AIDS status when developing reliable guidelines for the diagnosis, treatment, and prevention of bone fragility. Based on heterogeneity and potential bias in the previous studies, an individualized approach in the clinical assessment of skeletal alteration in PLWHIV is mandatory to address covariant effects of other comorbidities effectively.

## 4. Antiretroviral Therapy Effects on Osteodensitometry Findings in PLWHIV

Skeletal alterations associated with cART have been a significant concern for years, given that a substantially increased risk of low BMD was noted in individuals on cART compared to treatment-naïve individuals [20,23,24]. A summary of the main cART-induced bone effects is given in Table 1.

In short, initial pioneering studies reached contrary conclusions regarding the negative bone effects of cART containing protease inhibitors (PIs, Table 1) [16,23,26,31,32]. Also, several previous studies reported that cART based on tenofovir disoproxil fumarate (TDF) was associated with a low BMD [24,33,34,35,40]. The recent meta-analysis revealed that the widely available TDF regimen was associated with a substantial decrease in bone mass compared to other cART regimens used for pre-exposure prophylaxis and treatment of active HIV infection [35]. Bone loss associated with TDF use could at least be partially explained by its negative effect on kidney function (TDF-induced Fanconi syndrome and phosphaturia) [24,41,42], which was reported to be successfully restored upon TDF discontinuation and switching to tenofovir alafenamide (TAF)-based cART regimens [24,36,37,39]. These data were incorporated in the newest European AIDS Clinical Society guidelines, recommending TAF as the first-choice therapy over TDF for PLWHIV with skeletal alterations and kidney diseases [43].

Still, it is important to note that even though low BMD was noted, the TDF usage was not accompanied by a significant fracture risk increase [35], revealing the major limitation of BMD as a sole bone fragility determinant. It is indicative that this two-dimensional X-ray imaging factor cannot solely explain bone fragility, meaning that other bone properties should be investigated to complete the bone fragility puzzle in PLWHIV. This reasoning is supported by previous reports on the improvement of altered bone mechanical properties after switching from a TDF- to a TAF-based cART, which was independent of significant BMD alterations [44]. Moreover, alcohol abuse was reported to increase the risk of bone fractures in PLWHIV [24,45,46], but without a significant association between alcohol abuse and changes in vertebral or femoral BMD [47]. This suggests that the relationship between alcohol abuse and bone health, even in PLWHIV, could be influenced by factors not solely related to changes in BMD [48], warranting further detailed research.

## 5. The Importance of Multi-Scale Bone Assessment in PLWHIV

**Figure 1:**
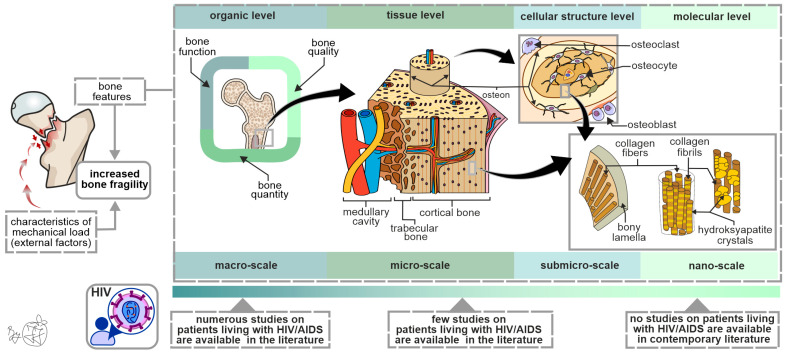
Bone strength determinants in PLWHIV: the importance of the multi-scale approach in the assessment of bone hierarchical organization. Since low-intensity force (a fall from standing height) is not sufficient to fracture a healthy bone, this is indicative that the main cause of increased bone fragility originates from the structural bone features. It is important to note that the contemporary literature contains limited data about submicro- and nano-scale bone properties in PLWHIV, warranting further research.

Since bone fracture occurrence is primarily dependent on characteristics of the mechanical load (external factors), bone properties (intrinsic factors), and their mutual interaction (Figure 1), it is evident that increased susceptibility to bone fractures cannot be fully explained by alterations in DXA-assessed BMD values. It is known that a considerable number of individuals with bone fractures have physiological BMD values [49], meaning that other bone properties (on various levels of a hierarchical bone organization, Figure 1) should be investigated to fully understand all factors that contribute to increased bone fragility in PLWHIV [50]. Moreover, BMD measurement is of limited informative value in individualized fracture risk assessment [51], highlighting the need for research on other bone fragility determinants in PLWHIV. The importance of applying a hierarchical approach in assessing bone properties is highlighted by the fact that some therapy regimens improved bone strength and reduced the fracture risk without being accompanied by an adequately increasing BMD [52,53,54].

Several attempts have been made to overcome the limitations of exclusively relying on DXA-generated BMD in clinical fracture risk assessment, one of which is known as the trabecular bone score (TBS) [55]. This gray-level textural measurement uses high-quality DXA images to indirectly estimate the lumbar micro-architecture (L1–L4). Recent studies revealed that PLWHIV are less likely to have normal TBS values [56]. Moreover, sub-clinical vertebral fracture occurrence was recently reported to be associated with TBS values in HIV-infected patients [57]. Also, loss of lean mass in PLWHIV was associated with lower TBS values, implying its potential as a therapeutic target for improving aging-associated bone strength decline [56]. Still, TBS has a major disadvantage due to its applicability to only one skeletal site and since it is an indirect measure of bone micro-architecture. To overcome these limitations, high-resolution peripheral quantitative computed tomography (HR-pQCT) was developed to allow a noninvasive 3D method for the clinical assessment of the bone micro-architecture at the distal radius and tibia. As shown in Table 2, the contemporary literature suggests that PLWHIV can display a range of micro-architectural alterations, which can contribute to site-specific bone strength decline in these individuals [55,58,59,60,61,62,63,64,65,66]. Namely, the most prominent alterations were noted in the distal tibia compared to the distal radius of PLWHIV. The differences in previous data about bone micro-architectural changes in PLWHIV may have originated from the study design (and associated bias of inclusion and exclusion criteria), the number of participants included in the study, the sex and age of the participants, the use of different cART regimes, and the presence of covariant bone-affecting comorbidities (Table 2). Also, the reliable applicability of HRpQCT is limited by its high costs and by the inability to access other clinically relevant fracture sites (e.g., proximal femora), indicating the need to use other state-of-the-art methodologies to further investigate other bone fragility determinants in PLWHIV [50]. Also, distinguishing the potential differences between trabecular and cortical bones and their contribution to the bone fragility of PLWHIV should be thoroughly pursued in the future using high-resolution imaging approaches [67].

Considering the lack of data in the contemporary literature (Figure 1), future studies should focus on morpho-structural and functional assessment of the osteocyte lacunar network, functional assessment of other bone cells, morpho-structural assessment of mineral and organic components of the bone extracellular matrix, and functional assessment of bone marrow adiposity to elucidate its role in increased bone fragility among PLWHIV. The informative value of these studies could be improved by utilizing multiple state-of-the-art methods to analyze various bone features within the same bone specimen from each individual patient [68]. Finally, by integrating clinical data, the hierarchical approach in bone assessment could lead to the development of patient-specific diagnostic algorithms for predicting bone strength in PLWHIV.

**Table 2:** Contemporary studies on bone micro-architectural alterations in PLWHIV.

Study (Reference)	Study Design	Number of Patients with HIV	Imaging Method	Assessed Skeletal Site	Main Results on Bone Micro-Architecture
Serrano S. et al. [58]	Case–control study	*n* = 22 male, *n* = 13 female, *n* = 9	Optic microscopy	Iliac bone	No significant difference in BVTV, Tb.Th, or Tb.N; significantly reduced osteoid volume and mildly altered osteoblast activity in PLWHIV.
Yin M. et al. [59]	Case–control study	*n* = 46 female, *n* = 46 on ART, *n* = 37	HRpQCT	DR, DT	No significant difference in trabecular or cortical micro-architecture in DR; reduced tibial Ct.Th was noted in postmenopausal WLWHIV; cART did not display a significant effect on bone micro-architecture.
Calmy A. et al. [60]	Case–control study	*n* = 22 female, *n* = 22 on ART, *n* = 22	HRpQCT	DR, DT	No significant difference in radial micro-architecture; low tibial Tb.N and high tibial Tb.Sp were noted in premenopausal WLWHIV; cART did not display a significant effect on bone micro-architecture.
Biver E. et al. [61]	Case–control study	*n* = 28 male, *n* = 28 on ART, *n* = 28	HRpQCT	DR, DT	Significantly low radial Tb.N and Ct.Th, coupled with high radial Tb.Sp, were noted in PLWHIV; reduced tibial Tb.Th was noted in men older than 60 years with long-term HIV infection.
Lo Re V. et al. [62]	Case–control study	*n* = 100 female, *n* = 100 HCV/HIV, *n* = 50	HRpQCT	DT	Low tibial Ct.Th was noted in WLWHIV, while tibial trabecular density and Ct.Th were lower in individuals with HCV/HIV confection.
Sellier P et al. [63]	Case–control study	*n* = 100 male, *n* = 53 on TDF, *n* = 53	HRpQCT	DR, DT	Trabecular micro-architecture deteriorated, while no significant changes were noted in the cortical compartment of PLWHIV treated with TDF.
Tan D. et al. [55]	Case–control study	*n* = 46 male, *n* = 36 with fracture, *n* = 23	HRpQCT	DR, DT	PLWHIV with prior bone fracture had a lower tibial trabecular bone mass and Ct.Th, coupled with a mild trend toward higher radial cortical porosity.
Kazakia G. et al. [64]	Case–control study	*n* = 8 male, *n* = 8 on ART, *n* = 8	MRI HRpQCT	PF, DR, DT	Lower Tb.Th and Tb.N of the femoral head, coupled with lower tibial Tb.N and higher tibial Tb.Sp in PLWHIV, compared to uninfected controls.
Foreman S. et al. [65]	Cross-sectional study	*n* = 43 male, *n* = 37 on ART, *n* = 43	HRpQCT	UDR, UDT	Malnutrition, physical activity, longer duration of HIV infection, and use of the TDF/PI combination were associated with an altered bone micro-architecture in PLWHIV.
MacDonald H. et al. [66]	Case–control study	*n* = 50 female, *n* = 50 on ART, *n* = 50	HRpQCT	DR, DT	Lower radial Tb.N and Tb.Th, coupled with lower tibial Tb.Th, were noted in WLWHIV. Tenofovir treatment may contribute to these bone deficits.
Shiau S. et al. [69]	Case–control study	*n* = 172 boys, *n* = 86 on ART, *n* = 172	pQCT	DR, DT	Reduced trabecular area, Ct.Th, and periosteal cortical circumference were noted in children living with HIV compared to uninfected controls.

Abbreviations: DR—distal radius; DT—distal tibia; PF—proximal femur; UDR—ultra-distal radius; UDT—ultra-distal tibia; BV/TV—bone volume/tissue volume; Tb.Th—trabecular thickness; Tb.N—trabecular number; Tb.Sp—trabecular separation; Ct.Th—cortical thickness; HRpQCT— high-resolution peripheral quantitative computed tomography; cART—combined antiretroviral therapy; TDF—tenofovir disoproxil fumarate; PI—protease inhibitor; PLWHIV—people living with HIV; WLWHIV—women living with HIV.

## 6. The Molecular Mechanisms Involved in Etiopathogenesis of Skeletal Alterations in PLWHIV

**Figure 2:**
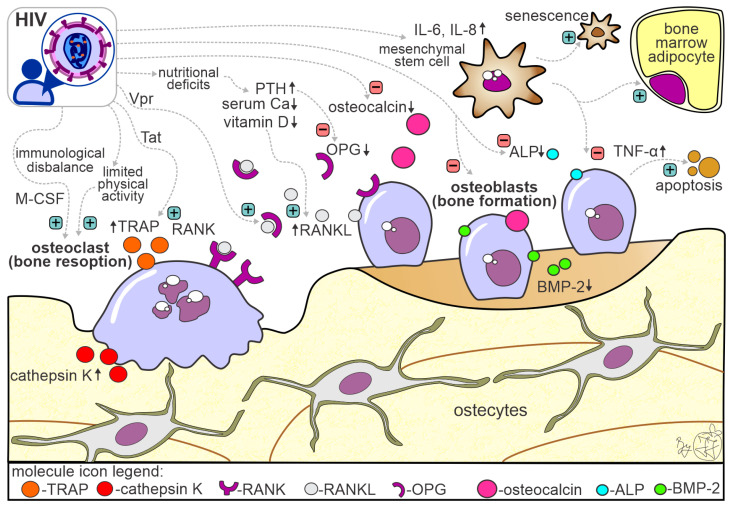
Schematic representation of molecular mechanisms contributing to bone alterations in patients living with HIV/AIDS. The figure contains multiple factors leading to bone changes in PLWHIV, with an emphasis on the factors that cause reduced bone formation and factors that stimulate bone resorption (the activating effect is marked with a green sign, while the deactivating effect is marked with a red sign; upward arrow symbol indicate higher concentrations, while downward arrow symbol indicate lower concentration of the molecule). Abbreviations: PTH—parathyroid hormone; IL—interleukin; TNF-α—tumor necrosis factor-α; ALP—alkaline phosphatase; OC—osteocalcin; OPG—osteoprotegerin; Vpr—viral protein R; M-CSF—macrophage colony-stimulating factor; Tat—trans-activator of transcription; RANK—receptor activator for nuclear factor-kappa B; RANKL—receptor activator for nuclear factor kappa B ligand; BMP-2—bone morphogenic factor-2.

Etiopathogenetic mechanisms underlying bone alterations in PLWHIV are complex and not fully understood (Figure 2). It is also important to note that bone alterations could be associated with the direct effect of HIV infection per se, with the direct or indirect toxic effect of cART, and with the indirect effect of other well-known confounding bone-affecting factors (e.g., aging, postmenopausal hormonal changes, body weight) [70]. It is known that bone loss in PLWHIV results from the complex interplay between immunological disbalance, cytokine disruptions, nutritional deficiencies, low serum calcium and vitamin D levels, hypogonadism and other hormonal disturbances, liver and kidney dysfunction, and low levels of physical activity/immobilization (Figure 2) [10,13,24,71].

Initial pioneering studies reached contrary conclusions regarding the possibility that HIV could affect bone cells and display cytopathic effects [72,73]. However, more recent studies revealed that HIV trans-activator of transcription (Tat) and negative regulatory factor (Nef) are associated with reduced osteoblastic differentiation of bone marrow mesenchymal stem cells (Figure 2), causing a predominant differentiation to the adipocyte lineage [71,74]. Also, these HIV proteins are shown to induce senescence of bone marrow mesenchymal stem cells through nuclear factor-κB pathway activation and inhibition of autophagy [74,75]. Furthermore, HIV proteins can trigger in vitro osteoblast apoptosis mediated by the up-regulation of tumor necrosis factor-α (TNF-α), which may be associated with reduced bone formation [71,76,77]. The direct HIV interference with bone formation is heightened by altered calcium deposition and alkaline phosphatase activity and by reduced levels of bone morphogenetic protein-2 (BMP-2) [78,79]. Furthermore, altered osteocalcin and sclerostin levels were suggested to contribute to bone alterations noted in PLWHIV [80,81]. On the other hand, HIV proteins Tat and Vpr increase monocyte differentiation into osteoclasts [82,83], as well as boost bone resorption through increased expression of RANKL and lower expression of osteoprotegerin (OPG) [71,84]. A positive feedback loop exists between RANKL production and HIV replication, which may be relevant to bone loss in PLWHIV (Figure 2). Also, it has been noted that the altered serum RANKL/OPG ratio contributes to skeletal abnormalities in PLWHIV compared to non-infected individuals [85]. Persistent activation of pro-inflammatory cytokines (TNF-α, interleukin-1—IL-1, interleukin-6—IL-6,) has an activation effect predominantly on bone resorption in PLWHIV [86,87]. It is important to note that this hyperinflammatory effect on increased bone resorption is amplified in individuals with liver disease due to HCV coinfection [70,88]. Moreover, components of metabolic syndrome, dyslipidemia, metabolic-associated fatty liver disease, type 2 diabetes mellitus, and insulin resistance have negative effects on bone turnover, contributing to bone loss in individuals living with HIV/AIDS [89,90].

Other factors that may contribute to skeletal alterations in PLWHIV are associated with alcohol abuse, use of opioids, heroin, or other illicit drugs, corticosteroid use, and cART use [13,91,92]. The cART-associated effect on etiopathogenetic mechanisms of bone loss in PLWHIV was extensively elaborated elsewhere [13,79], but it is important to note that various cART regimens could display variable effects on bone health [93]. Recent studies suggested that PI has a predominant effect on an increased rate of bone remodeling [94]. Moreover, TDF-based cART regimens were reported to affect bone turnover through the reduction in extracellular adenosine levels, mediated by the inhibition of ATP release from bone cells, leading to predominant bone resorption [95]. In addition, TDF interferes with the binding of vitamin D with vitamin D-binding protein, reducing its availability for the production of the active form of vitamin D in the kidneys [95]. Lower vitamin D levels result in less calcium and phosphorus being absorbed in the intestines, which could be associated with higher levels of parathyroid hormone and subsequent bone resorption increase [95]. On the other hand, due to differences in pharmacokinetics, plasma concentrations of the active metabolite are lower in TAF-based cART regimens, meaning that TAF has been reported to have a better bone health safety profile [95]. There are a few data that suggest that tenofovir and indinavir have direct or indirect negative effects on osteoblast function (bone formation), while ritonavir has a negative effect on bone resorption via declining osteoclast differentiation [96,97,98], warranting further research.

Since our understanding of the multifactorial etiopathogenetic mechanisms responsible for declining bone health in PLWHIV is based on limited research data, future research should focus on using state-of-the-art methodologies to conduct human bone assessments that will fully resolve the bone fragility puzzle in these individuals. These new insights may lead to the development of new therapeutic modalities that will be specifically designed to mitigate the health burden associated with skeletal disorders in PLWHIV.

## 7. Conclusions

Skeletal alterations are common in PLWHIV. Numerous studies have contributed to our understanding of bone fragility determinants in PLWHIV, but countless ambiguities persist. More detailed research on bone properties (especially at the submicro- and nano-scale) is required to improve our understanding of bone fragility determinants in PLWHIV. Combined with the available clinical data, taking a hierarchical approach to evaluating structural bone properties could set the basis for developing a patient-specific diagnostic algorithm that will reliably predict the fracture risk in PLWHIV. Additionally, apart from general guidelines for good bone health, specific clinical guidelines for individualized prevention, diagnosis, and treatment of skeletal disorders in PLWHIV should be established and regularly implemented [43,99,100,101], especially in countries with resource-limited clinical settings.

## Figures and Tables

**Table 1:** The main findings of contemporary studies on the potential adverse effects of antiretroviral therapy on bone mineral density in patients living with HIV/AIDS.

Study (Reference)	Study Design	Number of Patients	Assessed Skeletal Site	Main Results on ART-Induced BMD Alterations
Moore A. et al. [25]	Case–control study	male, *n* = 221 NRTI, *n* = 42 PI, *n* = 147	Total body, LV	Low total body BMD was associated with low body weight prior to commencing cART, while low lumbar BMD was associated with increased lactate concentrations.
Carr A. et al. [26]	Cross-sectional study	*n* = 105 male, *n* = 75 NNRTI, *n* = 47	LV, PF, FN	Low BMD was associated with using a PI-based cART regimen. Age, sex, race, and smoking were not associated with skeletal outcomes.
Knobel H. et al. [27]	Cross-sectional study	*n* = 80 male, *n* = 58 PI, *n* = 37	LV, PF	Low BMD was noted in PLWHIV, but without a significant association with any of the specific cART regimens.
Bruera D. et al. [16]	Case–control study	*n* = 142 male, *n* = 113 PI, *n* = 42 non-PI, *n* = 36	Total body, LV, PF	Low BMD at all assessed skeletal sites was noted in PLWHIV, irrespective of the cART regimen used.
Fernandez-Rivera J. et al. [28]	Prospective study	*n* = 89 PI, *n* = *32* NNRTI, *n* = 15	LV, FN	Low BMD was associated with PI-based cART, low plasma albumin level, and male sex. Bone loss did not substantially progress after 1 year of continued therapy.
Amiel C. et al. [29]	Cross-sectional study	*n* = 148 PI, *n* = 49 non-PI, *n* = 51	LV, FN	Low BMD was noted in PLWHIV, predominantly related to the low body weight, irrespective of the cART regimen used.
Garcia Aparicio A. et al. [30]	Cross-sectional study	*n* = 30 PI, *n* = 17	LV, PF, FN	The use of PI-based cART regimens was not associated with BMD alterations, while vitamin D deficiency and hypogonadism contributed to bone loss in the included individuals.
Brown T. et al. [23]	Meta-analysis	*n* = 884 ART, *n* = 824 PI, *n* = 791	LV, PF	Use of cART and especially the use of PI-based cART regimens were associated with low BMD.
Arnsten J. et al. [31]	Cross-sectional study	*n* = 328 ART, *n* = 285 PI, *n* = 242	LV, FN	Low BMD at both analyzed skeletal sites was associated with HIV infection after adjusting for age, weight, race, testosterone level, and prednisone and illicit drug use, irrespective of the cART regimen.
Madeddu G. et al. [32]	Longitudinal study	*n* = 67 ART, *n* = 62 PI, *n* = 27	LV, PF	Low BMD was associated with cART use in PLWHIV. Bone alterations may persist over time and further worsen with accelerated turnover, particularly in patients receiving PI-based cART regimens.
Stellbrink H. et al. [33]	Multicenter longitudinal study	*n* = 328 TDF, *n* = 193	LV, PF	More prominent bone alterations were noted in patients treated with tenofovir–emtricitabine than in patients treated with abacavir–lamivudine.
McComsey G. et al. [34]	Randomized control trial	*n* = 328 male, *n* = 228	LV, PF	Patients treated with TDF-FTC had significantly lower BMD compared to patients treated with ABC-3TC. Patients treated with ATV/r had lower lumbar BMD but not femoral BMD in comparison to EFV-treated individuals.
Baranek B. et al. [35]	Meta-analysis	Total number of individuals not reported	LV, PF	TDF caused more substantial BMD loss compared to other ART regimens. The effect was less prominent if used for pre-exposure prophylaxis than as a treatment for PLWHIV.
Mills A. et al. [36]	Randomized control trial	*n* = 1448 TDF, *n* = 477 TAF, *n* = 959	LV, PF	Switching to a TAF-containing regimen from the TDF-based regimen was non-inferior for the maintenance of viral suppression and led to improved BMD and renal function.
Pozniak A. et al. [37]	Randomized control trial	*n* = 242	LV, PF	After switching to a TAF-based cART regimen, proteinuria, proximal renal tubular function, and BMD significantly improved over 48 weeks compared to individuals initially on TDF-based cART.
Hill A. et al. [38]	Meta-analysis	*n* = 811,134, male, *n* = 6732	LV, PF	TDF boosted with ritonavir or cobicistat was associated with higher risks of skeletal alterations and lower HIV RNA suppression rates when compared with TAF. In contrast, when ritonavir and cobicistat were not used, there were no efficacy differences between TAF and TDF, and marginal differences were noted in drug safety.
Walmsley S. et al. [39]	Randomized control trial	*n* = 34, female, *n* = 34	LV	The trend was noted toward increased lumbar BMD after a switch from a TDF-based cART regimen to a TAF-based cART regimen in perimenopausal and early postmenopausal WLWHIV.

Abbreviations: LV—lumbar vertebrae; PF—proximal femur; FN—femoral neck; cART—combined antiretroviral therapy; BMD—bone mineral density; PLWHIV—people living with HIV/AIDS; WLWHIV—women living with HIV/AIDS; NRTI—nucleoside analogue reverse transcriptase inhibitor; NNRTI—non-nucleoside reverse transcriptase inhibitor; PI—proteinase inhibitor; TDF-FTC—tenofovir disoproxil fumarate emtricitabine; ABC-3TC—abacavir–lamivudine; ATV/r—atazanavir–ritonavir; EFV—efavirenz; TDF—tenofovir disoproxil fumarate; TAF—tenofovir alafenamide.

## Data Availability

No new data were generated during this narrative literature review. The obtained literature search results supporting the claims in this narrative review can be made available from the corresponding author upon a justified request.
